# Influence of Gait Speeds on Contact Forces of Lower Limbs

**DOI:** 10.1155/2017/6375976

**Published:** 2017-07-09

**Authors:** Xin Wang, Yue Ma, Bo Yi Hou, Wing-Kai Lam

**Affiliations:** ^1^Department of Kinesiology, Shenyang Sport University, Shenyang 110102, China; ^2^Key Laboratory of Impression Evidence Examination and Identification Technology, Criminal Investigation Police University of China, Shenyang 110854, China; ^3^Ningwu Country Senior High School, Ningwu 036700, China; ^4^Li Ning Sports Science Research Centre, Beijing 101111, China

## Abstract

While walking with fast speed aims to promote health and fitness of individuals, the potential risk on lower limb joint loading across walking speed is still unknown. In order to determine the joint contact force loading associated with different walking speeds, fifteen young male and fifteen female participants performed barefoot walking across different speeds (regular = 1.1 m/s, medium = 1.4 m/s, and fast = 1.7 m/s). The synchronized motion and ground reaction force (GRF) data were captured by Codamotion capture system and AMTI force platform. All kinematics and GRF information were input to the AnyBody musculoskeletal model to determine 3-dimensional knee contact forces. The results showed that increased walking speed was associated with a greater proximal-distal and anterior-posterior GRF during early impact phase, implying that the joint stability is more demanding at higher walking speed conditions (*P* < 0.05). In addition, higher proximal-distal and anterior-posterior knee contact forces were found when participants were walking at higher speeds (*P* < 0.05). Therefore, the risk of knee cartilage and ligament damage associated with the increased knee contact forces should require further attention.

## 1. Introduction

Power walking or speed walking, which is defined as the walking with an individual's fastest speed, is a popular fitness exercises among cities in China. The aim of speed walking is to promote heart rate fitness and endurance of participants. However, most participants only concern about the walking speed, but pay little attention on the impact load on lower limbs, which may result in higher injury risks especially in the ankle or knee joint [1–3]. Landing movements during walking [4], running [5], gymnastics [6], volleyball [7], soccer [8], and Australian football [9] have been studied using with kinetic, kinematic, and electromyography parameters for the evaluation of injury risk or performance. In these studies, ground reaction forces (GRF) and the ankle and knee joint forces provide key and fundamental information to understand loading [10, 11]. Furthermore, these force parameters are often compared among different subject groups to identify biomechanical differences [12–14].

Regarding the research on walking biomechanics, the researchers [15, 16] have found that when walking at higher gait speeds, the walking kinematics and kinetics would be changed. Higher gait speeds were associated with larger step length, knee flexion angle, and peak plantar pressure, but with smaller ankle range of motion and shorter total contact times. In addition, other kinetic studies have shown that increased gait speed is related to greater peak plantar pressure and GRF [17–19]. Particularly, Sneyers et al. [20] found that the walking speed had significant impact on the foot pressure at the forefoot and rearfoot regions. Bertseh et al. [21] pointed out that distributing foot plantar pressure evenly can effectively reduce foot injuries. In the similar vein, too soft or hard, the interface material used may cause damage on the foot and affect performance.

In order to determine how GRF influences the risk of injury during landing or performance during push off in different movements, various methods have been used to show high correlations among GRF, lower limb kinematics, and related muscle activities, suggesting that the GRF has to be overcome or absorbed by musculature supporting of the ankle, knee, and hip joints [22, 23]. However, all of these kinetic and kinematic parameters should be integrated to evaluate the risk of injury and performance in a certain movement task. In addition, the GRF and knee contact forces are the common indicators for interpreting and explaining by the athletic trainers, scientists, and physicians. Furthermore, Haight et al. [15] compared peak tibiofemoral joint contact force (TF) when obese and nonobese participants walking at different speeds and on different slopes. Their results showed that at fast gait speeds, participants would effectively reduce the maximum TF at uphill walking compared with that at level walking. The TF was reduced by 23% (from 2352 N to 1811 N) and 35% (from 1994 N to 1303 N) for obese and nonobese participants, respectively. Nevertheless, TF is the resultant knee force (KF) exerted on the knee, and it is believed that 3D KF information can provide additional information for better estimation of knee joint loadings during walking at different speeds. Hence, the purpose of this study was to investigate joint kinematics, GRF, and KF in each of proximal-distal, anterior-posterior, and medial-lateral components when participants are walking at regular, medium, and fast paces.

## 2. Experimental Work

Fifteen young healthy male participants and fifteen female participants were recruited to perform five successful barefoot walking trials at different speeds (regular 1.1 m/s, medium 1.4 m/s, and fast 1.7 m/s) [4, 16]. The information of participants is listed in Table 1.

GRF were recorded at 1000 Hz using force plate (AMTI, Watertown, MA, USA) and synchronized motion data were captured at 250 Hz using Codamotion infrared capturing system. In order to minimize the body mass effect, all kinematics data were normalized with body mass. All the GRF and lower limb kinematics information were input to determine 3D knee joint forces using the AnyBody musculoskeletal model (AnyBody Modeling System v.6.0.3, Anybody Technology A/S, Aalborg, Denmark) in all walking speed conditions. The model consisted of the pelvis, legs, and feet and 35 leg muscles was built according to the previous studies [11–14]. All statistical analyses were performed with SPSS 19.0 (SPSS, Chicago, IL, USA). 2 (gender) × 3 (speed) mixed ANOVA were performed on gait parameters to determine if there was any interaction, gender, and speed effects. All data were presented as mean ± standard deviation. Significant level was set at *P* = 0.05. Since no gender effect was evident for force parameters, we pooled all subject data and performed one-way repeated measures ANOVA to assess the speed effect on each of the KF components.

## 3. Results

### 3.1. Kinematics Data

In Table 2, longer step length, faster stride frequency, and shorter stance time were observed at a faster walking speed, compared with those at regular and medium speed conditions (*P* < 0.05). Shorter stance, heel contact, forefoot, and toe-off times were found at fast speed compared with those at regular speed condition (*P* < 0.05). Regarding gender effect, male participants had longer step length (*P* < 0.01) and higher stride frequency compared with female participants (*P* < 0.01).

In Table 3, as walking speeds increased, larger dorsiflexion and smaller knee flexion were found at all contact phases (*P* < 0.05). At forefoot contact, knee flexion was decreased by 10° in fast speed compared with that in the other two speed conditions (*P* < 0.05). At toe-off, larger ankle and knee joint angles are at fast speed compared with those at regular speed (*P* < 0.01).

### 3.2. GRF Data

**Figure 1:**
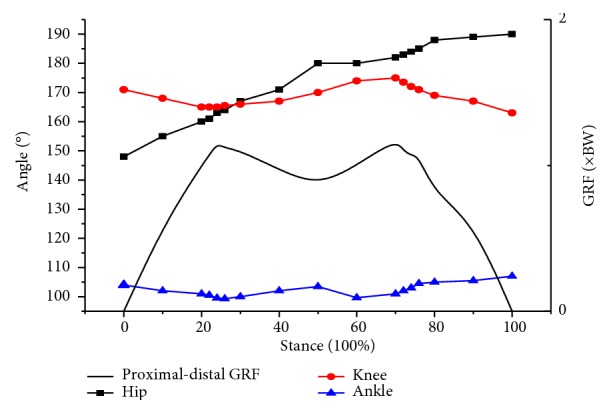
Typical joint angles and vertical GRF in regular speed.

Since no gender effect was evident for each GRF variable, we pooled all participant data to further assess the GRF components associated with walking speeds [7, 8]. In general, there are two peak curves shown for vertical GRF.  Figure 1() shows that first peak occurred at heel contact which was about 23 to 26% of stance. The second peak occurred at forefoot contact which was about 73 to 77% of the stance or maximum knee extension during take-off.

In Table 4, a greater first peak of vertical GRF was observed in fast speed condition compared with that in regular speed condition (*P* < 0.05), but no significant speed difference was found for the second peak of vertical GRF. In addition, higher walking speed resulted in higher anterior-posterior and medial-lateral GRF (*P* < 0.01).

### 3.3. 3D Knee Joint Contact Forces

**Figure 2:**
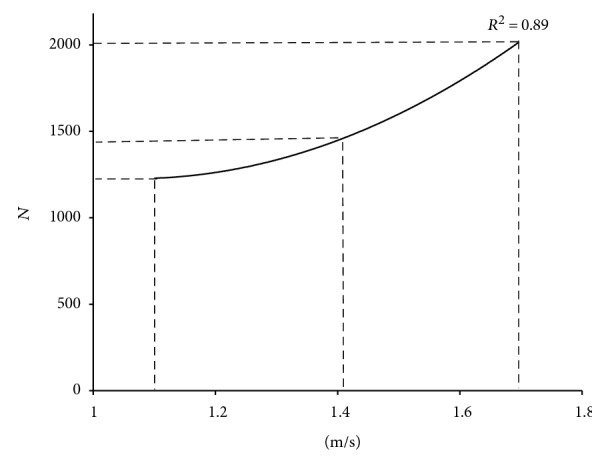
Relationship between proximal-distal KF across walking speed.

Table 5 shows that participants walking at fast speed experienced higher both proximal-distal and anterior-posterior KFs during heel contact phase compared to those at regular and medium speed conditions (*P* < 0.01). However, medial-lateral KF was not different among speed conditions (*P* > 0.05). The proximal-distal KF was increased by 20.75% and 64.62% when compared to that of medium and fast speeds, respectively. However, there was no main effect of speed in 3D KF at toe-off phase (*P* > 0.05). Regarding the proximal-distal KF, we further calculated the proximal-distal KF the relationship between proximal-distal KF and walking speed using polynomial fitting (*R*^2^ = 0.89). The regression model (Figure 2()) shows that when walking speed was below 1.2 m/s, only gentle change of the KF would be observed; when the speed exceeded 1.4 m/s, the KF would increase rapidly.

(1) $$\begin{array}{c} Y=1983.3X^{2}-4228.3X+3480.3. \end{array}$$

## 4. Discussion

This study investigated kinematics, GRF, and KF when participants were walking at regular, medium, and fast paces. Our results showed that increasing of speed was associated with higher strike frequency, shorter stance time, and increased the vertical and anterior-posterior GRF during impacts. To attenuate the impact forces, one body would elicit larger knee and ankle joint flexion at high walking speed condition compared with those at regular speed condition. In addition, increased knee extension angle during toe-off was found in high speed compared with that in regular speed condition. The extended knee angle and short stance time are thought to be beneficial to generate more muscle power to push body forward in a fast walking pace. Previous studies [19, 27] compared the EMG activation pattern (onset time and magnitude) of hamstring and semimembranosus during different walking conditions. They suggested that at early stance, the greater semimembranosus EMG was found in uphill walking compared with that in level walking and that the estimated muscle forces were increased across the walking speeds.

Regarding GRF in walking, the current results indicated that the vertical GRF was greater than anterior-posterior or medial-lateral GRF, which is in line with most research on walking. Typically, there are two peaks in vertical GRF curve [1]. The first peak force (F_1_) is produced by the impact of the heel and is always of lower magnitude than that of the second peak force (F_3_), which occurs at forefoot contact phase [3]. However it remains debating how GRF peaks would influence the risk of impact injury on lower limbs. It is likely that increased first impact GRF peak is related to plantar load on the heel. For regular walking speed, about 3.3 kg/cm^2^ of the plantar load can be absorbed by heel pad [28]. Plantar loads increase with walking speed. When one person walks with a long period of time, fat at heel pad is gradually shrinking and the heel and foot may be susceptible to damage. During the toe-off phase, knee and ankle extension have to be increased for better transfer of muscular power [29]. The current results indicated that increased walking speed has little influence on vertical GRF (F_3_), but has impact anterior-posterior GRF that facilitate faster forward movement.

In addition, the present findings showed the maximum knee contact force had two obvious peaks, which supported the findings measured using embedded sensors [24, 25]. In the present study, at fast walking speed, the proximal-distal and anterior-posterior KF were above three and one times body weight, respectively. These results are in line with a previous study [3, 25], which had obese and nonobese participants walking at fast speed (1.75 m/s) and at slow speed uphill (0.75 m/s, 6° inclined surface) and showed the peak TF was about 3.12 BW. In addition, the maximum knee contact force (2645 N or 3.0 BW) was occurred in 40% of the contact phase [30, 31]. Furthermore, KF become larger across walking speeds regardless of the obese or nonobese participants [15]. Considering the skeletal muscle system may not be fast enough to react and attenuate impact forces effectively, the participants may be exposed to higher risk of knee joint injury.

When interpreting our results, it is important to consider several limitations in our study. First, only young participants were recruited and hence our kinematics findings may not be applicable to the older adults. Second, the surface EMG data were not matched with actual activation of specific lower extremity muscles. A needle EMG or other techniques should be used to explain the change of activity of the leg muscles in different walking speed conditions. Future study should generalize the relationship among 3D knee contact forces, muscle cocontraction, and joint kinematics during walking in different age populations.

## 5. Conclusions

Fast walking is characterized as longer step length, faster step frequency, and shorter stance time. Participants walking at higher speed exhibited greater vertical and anterior-posterior GRF during stance phase, which may challenge walking stability and therefore elicited higher knee joint contact forces. Although fast walking is encouraged to build up fitness, the potential risk of knee cartilage and ligament injuries associated with increased knee contact forces should need further attention.

## Figures and Tables

**Table 1:** Participant information.

	Male (*n* = 15)	Female (*n* = 15)
Age (years)	24.6 ± 1.19	24.8 ± 1.13
Height (m)	1.76 ± 0.02	1.64 ± 0.02
Weight (kg)	68.3 ± 1.72	54.0 ± 1.92
BMI (kg/m^2^)	21.8 ± 0.34	20.0 ± 0.41
Shoe size (UK)	42.0 ± 0.00	37.0 ± 0.00

**Table 2:** Temporal-distance parameters change of stride across different speed conditions.

	Gender	Regular (1.1 m/s)	Medium (1.4 m/s)	Fast (1.7 m/s)
Step length (m)	Male	1.20 ± 0.09	1.30 ± 0.13^△^	1.50 ± 0.11^∗∗^
	Female	1.14 ± 0.10	1.21 ± 0.14	1.30 ± 0.17^

Stride frequency	Male	98.21 ± 9.37	114.45 ± 8.21^△△^	131.12 ± 10.22^∗∗^^^
	Female	104.12 ± 10.48	125.42 ± 14.26^△△^^	141.14 ± 9.12^∗∗^^^

Stance time (%)	Male	59.86 ± 3.13	57.31 ± 4.75^△^	54.34 ± 4.06^∗∗^
	Female	60.21 ± 2.99	56.82 ± 2.79^△^	54.99 ± 3.53^∗∗^

Heel contact time (%)	Male	15.49 ± 5.31	17.56 ± 5.13	19.32 ± 5.76^∗^
	Female	15.29 ± 6.22	18.90 ± 3.53	20.13 ± 4.21^∗^

Forefoot contact time (%)	Male	17.29 ± 4.99	15.30 ± 4.72	14.86 ± 4.32
	Female	18.22 ± 3.15	16.14 ± 4.68	15.86 ± 4.15

Toe-off time (%)	Male	33.32 ± 4.21	35.74 ± 4.32	37.35 ± 6.76^∗^
	Female	31.68 ± 7.88	34.15 ± 6.07	35.99 ± 4.13^∗^

∗ means the significant difference between regular and fast speeds. △ means the significant difference between medium and fast speeds. ^ means the significant difference between genders. ∗∗ means the significant difference between regular and fast speeds and *p* < 0.01. △△ means the significant difference between medium and fast speeds and *p* < 0.01. ^^ means the significant difference between genders and *p* < 0.01.

**Table 3:** Ankle and knee joint angle positions at different contact phases across different speed conditions.

	Gender	Regular (1.1 m/s)		Medium (1.4 m/s)		Fast (1.7 m/s)	
		Ankle	Knee	Ankle	Knee	Ankle	Knee
At heel contact	Male	104.01 ± 6.21	171.36 ± 7.23	102.31 ± 6.23	169.49 ± 7.43	99.68 ± 5.57^∗^	168.80 ± 7.28
	Female	103.45 ± 7.31	172.64 ± 8.23	100.35 ± 5.14	168.15 ± 4.77	98.18 ± 8.65^∗^	168.24 ± 70.42

At forefoot contact	Male	99.50 ± 5.23	164.98 ± 6.74	99.52 ± 6.21	160.56 ± 5.45^△^	97.66 ± 4.32	155.70 ± 6.12^∗∗^
	Female	98.12 ± 6.22	163.67 ± 3.11	97.32 ± 2.71	158.45 ± 6.89^△^	96.33 ± 3.19	156.33 ± 4.12^∗∗^

At heel off	Male	93.67 ± 4.25	171.55 ± 6.54	94.32 ± 3.25	170.37 ± 6.47	97.05 ± 5.12^∗^	169.72 ± 6.54
	Female	91.22 ± 5.12	172.01 ± 4.62	92.22 ± 4.12	169.99 ± 4.89	95.48 ± 5.14^∗^	169.34 ± 5.31

At toe-off	Male	108.60 ± 8.21	153.78 ± 6.54	111.65 ± 7.23	155.36 ± 7.56^△^	116.58 ± 9.12^∗^	159.43 ± 6.32^∗∗^
	Female	107.2 ± 7.35	154.43 ± 7.45	109.31 ± 4.77	156.33 ± 8.45^△^	115.22 ± 7.23^∗^	160.47 ± 6.77^∗∗^

∗ means the significant difference between regular and fast speeds. △ means the significant difference between medium and fast speeds. ∗∗ means the significant difference between regular and fast speeds and *p* < 0.01.

**Table 4:** Vertical GRF across different walking speeds.

		Regular (1.1 m/s)	Medium (1.4 m/s)	Fast (1.7 m/s)
Vertical GRF (BW)	First peak (F_1_)	1.17 ± 0.44^#^	1.37 ± 0.42	1.52 ± 0.53^∗^
	Minimum (F_2_)	0.86 ± 0.22	0.76 ± 0.23	0.62 ± 0.26^∗^
	Second peak (F_3_)	1.10 ± 0.23	1.08 ± 0.18	1.09 ± 0.16

Anterior-posterior GRF (BW)	First minimum (F_4_)	−0.08 ± 0.03	−0.10 ± 0.06^△^	−0.13 ± 0.05^∗∗^
	Peak (F_5_)	0.17 ± 0.04	0.19 ± 0.05^△^	0.22 ± 0.05^∗∗^
	Second minimum (F_6_)	−0.23 ± 0.06	−0.25 ± 0.05	−0.27 ± 0.07^∗^

Medial-lateral GRF (BW)	First peak (F_7_)	0.06 ± 0.03	0.06 ± 0.04^△^	0.08 ± 0.03^∗^
	Second peak (F_8_)	0.04 ± 0.03	0.05 ± 0.03^△^	0.07 ± 0.04^∗∗^

∗ means the significant difference between regular and fast speeds. △ means the significant difference between medium and fast speeds. ∗∗ refers to the significant difference between regular and fast speeds and *p* < 0.01. # refers to the significant difference between regular and medium speeds and *p* < 0.05.

**Table 5:** Peak knee contact forces (KF) across different walking speeds.

		Regular (1.1 m/s)	Medium (1.4 m/s)	Fast (1.7 m/s)
Heel contact	Proximal-distal KF (BW)	2.12 ± 0.51	2.56 ± 0.48^△△^	3.49 ± 0.53^∗∗^
	Anterior-posterior KF (BW)	0.70 ± 0.15	0.81 ± 0.17^△△^	1.23 ± 0.26^∗∗^
	Medial-lateral KF (BW)	0.43 ± 0.09	0.45 ± 0.12	0.45 ± 0.10

Toe-off	Proximal-distal KF (BW)	3.39 ± 0.57	3.39 ± 0.49	3.41 ± 0.54
	Anterior-posterior KF (BW)	1.06 ± 0.13	1.08 ± 0.17	1.11 ± 0.26
	Medial-lateral KF (BW)	0.72 ± 0.13	0.73 ± 0.12	0.75 ± 0.16

∗∗ means the significant difference between regular and fast speeds and *p* < 0.01. △△ means the significant difference between medium and fast speeds and *p* < 0.01.
