# Erratum to “Influence of Gait Speeds on Contact Forces of Lower Limbs”

**DOI:** 10.1155/2018/9291423

**Published:** 2018-07-18

**Authors:** Xin Wang, Yue Ma, Bo Yi Hou, Wing-Kai Lam

**Affiliations:** ^1^Department of Kinesiology, Shenyang Sport University, Shenyang 110102, China; ^2^Key Laboratory of Impression Evidence Examination and Identification Technology, Criminal Investigation Police University of China, Shenyang 110854, China; ^3^Ningwu Country Senior High School, Ningwu 036700, China; ^4^Li Ning Sports Science Research Centre, Beijing 101111, China

In the article titled “Influence of Gait Speeds on Contact Forces of Lower Limbs” [1], references [15] and [25] were duplicated, and reference [15] had incorrect details. Therefore, reference [15] should be corrected as follows: “D. J. Haight, Z. F. Lerner, W. J. Board, and R. C. Browning, “A comparison of slow, uphill and fast, level walking on lower extremity biomechanics and tibiofemoral joint loading in obese and nonobese adults,” *Journal of Orthopaedic Research*, vol. 32, no. 2, pp. 324–330, 2014.”

Additionally, there were errors in the Discussion. The corrected section and its corresponding references are as follows:

## 4. Discussion

This study investigated kinematics, GRF, and KF when participants were walking at regular, medium, and fast paces. Our results showed that increased speed was associated with higher strike frequency, shorter stance time, and increased the vertical and anterior-posterior GRF during impact. To attenuate the impact forces, one body would elicit larger knee and ankle joint flexion at the high walking speed condition compared with the regular speed condition. In addition, increased knee extension angle during toe-off was found in the high speed compared with the regular speed condition. An extended knee angle and short stance time are thought to be beneficial to generate more muscle power to push the body forward in a fast walking pace. Previous studies [15, 19] compared the EMG activation pattern (onset time and magnitude) of hamstring and semimembranosus during different walking conditions. They suggested that, at early stance, the greater semimembranosus EMG was found in uphill walking compared with level walking and that the estimated muscle forces were increased across the walking speeds.

Regarding GRF in walking, the current results indicated that the vertical GRF is greater than anterior-posterior or medial-lateral GRF, which is in line with most research on walking. Typically, there are two peaks in the vertical GRF curve [1]. The first peak force (F_1_) is produced by the impact of the heel and is always of a lower magnitude than the second peak force (F_3_), which occurs at the forefoot contact phase [3]. However, it remains debatable how GRF peaks would influence the risk of impact injury on lower limbs. It is likely that an increased first impact GRF peak is related to a plantar load on the heel. For normal walking speed, about 3.3 kg/cm^2^ of the plantar load can be absorbed by the heel pad [26]. Plantar loads increase with walking speed. When one person walks with for a long period of time, fat in the heel pad gradually shrinks, and the heel and foot may be susceptible to damage. During the toe-off phase, knee and ankle extension have to be increased for a better transfer of muscular power [27]. The current results indicated that increased walking speed has little influence on vertical GRF (F_3_) but has an impact on anterior-posterior GRF that facilitate faster forward movement.

In addition, the present findings showed the maximum knee contact force that had two obvious peaks, which supported the findings measured using embedded sensors [15, 24]. In the present study, at fast walking speeds, the proximal-distal and anterior-posterior KF were above 3 and 1 times the body weight, respectively. These results are in line with previous studies [3, 15], which had obese and nonobese participants walking at a fast speed (1.75 m/s) and at a slow speed uphill (0.75 m/s, 6° inclined surface) and showed the peak TF was about 3.12 BW. In addition, the maximum knee contact force (2645 N or 3.0 BW) occurred in 40% of the contact phase [28]. Furthermore, KF becomes larger across walking speeds regardless of the participants being obese or not [15]. Considering that skeletal muscle system may not be fast enough to react and attenuate impact forces effectively, the participants may be exposed to higher risk of knee joint injury.

When interpreting our results, it is important to consider several limitations in our study. First, only young participants were recruited and hence our kinematics findings may not be applicable to older adults. Second, the surface EMG data were not matched with actual activation of specific lower extremity muscles. A needle EMG or other techniques should be used to explain the change of activity of leg muscles in different walking speed conditions. Future studies should generalize the relationship among three-dimensional knee contact forces, muscle cocontraction, and joint kinematics during walking in different age populations.

## References


  [15] D. J. Haight, Z. F. Lerner, W. J. Board et al., “A comparison of slow, uphill and fast, level walking on lower extremity biomechanics and tibiofemoral joint loading in obese and nonobese adults,” *Journal of Orthopaedic Research*, vol. 32, no. 2, pp. 324–330, 2014.  [24] S. F. Scanlan, A. M. Chaudhari, C. O. Dyrby, and T. P. Andriacchi, “Differences in tibial rotation during walking in ACL reconstructed and healthy contralateral knees,” *Journal of Biomechanics*, vol. 43, no. 9, pp. 1817–1822, 2010.  [25] L. Lohmander, A. Östenberg, M. Englund, and H. Roos, “High prevalence of knee osteoarthritis, pain, and functional limitations in female soccer players twelve years after anterior cruciate ligament injury,” *Arthritis & Rheumatism*, vol. 50, no. 10, pp. 3145–3152, 2004.  [26] R. Hosein and M. Lord, “A study of in-shoe plantar shear in normal,” *Clinical Biomechanics*, vol. 15, no. 1, pp. 46–53, 2000.  [27] I. J. Ho, Y. Y. Hou, C. H. Yang, W. L. Wu, S. K. Chen, and L. Y. Guo, “Comparison of plantar pressure distribution between different speed and incline during treadmill jogging,” *Journal of Sports Science and Medicine*, vol. 9, no. 1, pp. 154–160, 2010.  [28] G. Scott, H. B. Menz, and L. Newcombe, “Age-related differences in foot structure and function,” *Gait & Posture*, vol. 26, no. 1, pp. 68–75, 2007.

